# TElomeric repeat-containing RNA (TERRA): Physiological functions and relevance in cancer

**DOI:** 10.3389/fonc.2022.913314

**Published:** 2022-08-02

**Authors:** Michal Kroupa, Kristyna Tomasova, Miriam Kavec, Pavel Skrobanek, Tomas Buchler, Rajiv Kumar, Ludmila Vodickova, Pavel Vodicka

**Affiliations:** ^1^ Department of the Molecular Biology of Cancer, Institute of Experimental Medicine of the Czech Academy of Sciences, Prague, Czechia; ^2^ Faculty of Medicine and Biomedical Center in Pilsen, Charles University, Pilsen, Czechia; ^3^ Department of Oncology, First Faculty of Medicine, Charles University and Thomayer University Hospital, Prague, Czechia; ^4^ Division of Molecular Genetic Epidemiology, German Cancer Research Center, Heidelberg, Germany; ^5^ Institute of Biology and Medical Genetics, First Faculty of Medicine, Charles University, Prague, Czechia

**Keywords:** TERRA, long non-coding RNA, telomere homeostasis, cancer, Alternative lengthening of telomeres

## Abstract

Telomeres are complex protective structures located at the ends of linear eukaryotic chromosomes. Their purpose is to prevent genomic instability. Research progress in telomere biology during the past decades has identified a network of telomeric transcripts of which the best-studied is TElomeric Repeat-containing RNA (TERRA). TERRA was shown to be important not only for the preservation of telomere homeostasis and genomic stability but also for the expression of hundreds of genes across the human genome. These findings added a new level of complexity to telomere biology. Herein we provide insights on the telomere transcriptome, its relevance for proper telomere function, and its implications in human pathology. We also discuss possible clinical opportunities of exosomal telomere transcripts detection as a biomarker in cancer precision medicine.

## Introduction

### Telomeres

Human chromosomes end with telomeres, the structures comprised of hundreds to thousands of hexameric DNA repeats (5´-TTAGGGn-3´ in vertebrates) and terminated by a single-stranded guanine-rich (G-rich) overhang (1). Telomeres are approximately 6 to 20 kilobases long in humans (2), with considerable length heterogeneity between tissues of an individual and even between distinct chromosomes within a cell (3). Due to the abundance of guanine, telomeres facilitate the formation of the structures called “G-quadruplexes” where guanines alignments are stabilized by hydrogen bonds (4). Furthermore, telomeres form lariat-like structures called T- and D-loops by invading the 3´ single-stranded (ss)DNA overhang into the double-stranded telomeric site (5). These structures are indispensable for the proper function of telomeres, and their formation has to be strictly regulated during the cell cycle by proteins of the shelterin multimeric complex (6, 7).

Shelterin consists of six protein subunits, namely TRF1, TRF2, RAP1, TIN2, TPP1, and POT1 (8). TRF1 and TRF2 subunits are recruited to canonical double-stranded telomeric DNA (9). Both proteins, along with RAP1 are connected *via* the TIN2 protein bridge, which binds TPP1, an interacting partner of the POT1 shelterin subunit (10). POT1 has a high affinity to the 3´ ssDNA G-rich overhang. Overall, shelterin mediates the proper formation of telomeric chromatin following DNA replication (8). The key function of the shelterin complex is to assist the T-loop formation, repression of 5´ end hyper-resection, and avert inappropriate activation of DNA damage response (DDR) pathways at the ends of chromosomes (8). Emerging evidence indicates that the shelterin function and proper telomere homeostasis, in general, are regulated by telomere transcripts known as TERRA “TElomeric Repeat-containing RNA” (11).

Telomeres naturally become progressively shorter with each cell division due to the end-replication problem (7). Critically shortened telomeres elicit a DDR pathway which may trigger apoptosis or a replicative senescence state (12) also known as the M1 stage (13). Additionally, the accumulation of DNA damage at the ends of chromosomes was observed in non-dividing differentiated somatic cells (14) presumably due to the action of DNA damaging agents (15). Such DNA damage is accumulated in the form of Telomere-associated DDR foci (16). Bypass of the senescence, occurring for example *via* de-activation of tumor suppressors such as p53 (17), p21 (18), Rb (19), along with telomere uncapping potentially result in massive genomic instability, and ultimately in malignant transformation. To avoid apoptosis and acquire an immortal phenotype, premalignant cells have to stabilize telomeres *via* the reactivation of telomerase or by alternative lengthening of telomeres (ALT), the two pivotal telomere maintenance mechanisms (TMMs) (2, 20). Currently known TMMs have recently been shown to harbor distinct TERRA expression patterns (11). In the present article, we also discuss differences in TERRA expression between telomerase and ALT-positive tumors.

## TElomeric repeat-containing RNA (TERRA) and its functions

Telomeres were historically viewed as generally heterochromatic and thus creating a transcriptionally repressive chromatin environment (21). In 1989 Rudenko and Van der Ploeg identified a heterogeneous population of RNA transcripts containing telomeric repeats in protozoa (*Trypanosoma brucei*) (22). The evidence of telomeric transcription in mammals was provided in 2007 when Azzalin et al. discovered TERRA molecules in a human cervical cell line (HeLa) (23). As shown by northern blot and RT-PCR, telomeric and subtelomeric regions are actively transcribed into TERRA molecules which are made of subtelomeric-derived RNA and UUAGGG repeats (23). In 2008 Schoeftner and Blasco characterized TERRA molecules as a novel structural component of telomeric chromatin having the capacity to regulate telomerase activity (24).

TERRA is a long non-coding (lnc) RNA, with transcription starting in the subtelomeric regions and terminating within the region of telomeric repeats. The telomeric C-rich strand is utilized as a template for TERRA transcription (25). TERRA is heterogeneous in its length ranging from 100 bases up to 10 kilobases (16, 17), while the majority of TERRA contains a (UUAGGG)n telomeric repeat tract with an average length of 200 bases. Therefore, the length heterogeneity of TERRA is probably due to the subtelomere-derived sequences (25)

**Figure 1:**
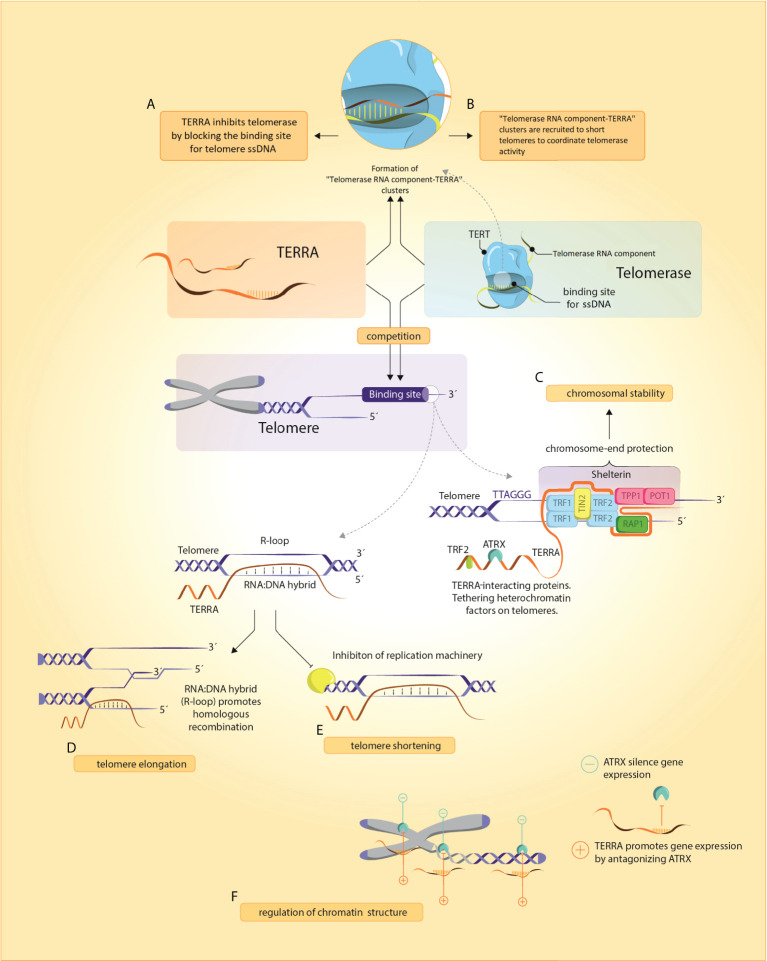
TERRA and its functions. **(A, B)** TERRA presumably binds to Telomerase RNA component through base-complementary pairing and blocks telomerase binding to telomeric ssDNA. In yeasts, Telomerase RNA component-TERRA clusters are localized at short telomeres where they coordinate telomerase activity, **(C)** TERRA and shelterin are implicated in chromosome-end protection by assembling secondary protective structures including R- and T-loops and G-quadruplexes **(D, E)** Regulation of telomeres by TERRA can induce either a shortening (by inhibition of telomerase activity and repressing *TERT* expression) or elongation (by homologous recombination promotion), **(F)** TERRA remodels chromatin structure through an antagonistic interaction with ATRX.

Deciphering the role of TERRA was a major unresolved question of telomere biology in the past decade as TERRA loci were unknown, preventing further functional studies (26) and because of unsuccessful efforts to fully deplete TERRA molecules (27). However, in 2017, new insights in a complex landscape of TERRA functions were achieved by successful degradation of TERRA *in vivo* (27). The following chapter summarizes the key roles of TERRA in physiological and pathological processes (summarized in **Figure 1**).

### TERRA as a regulator of chromatin structure

Though the specific functions of lncRNAs remain ill-defined, the molecules are linked with modulation of chromatin structure and recruitment of chromatin-modifying proteins to distinct genome regions. Based on the TERRA-protein interactome, an extensive network of TERRA-associated proteins has been identified in mouse embryonic stem cells (27). One of the crucial proteins identified in the study was ATRX, a chromatin remodeler frequently mutated in ALT-positive cancer cells. TERRA antagonizes ATRX localization at telomeric sites, having an impact on telomeric chromatin structure (27). Furthermore, TERRA and ATRX foci are not restricted to telomeres. TERRA and ATRX were shown to share genomic targets and modulate the expression of hundreds of genes across the genome (27), such as downregulation of genes involved in TOR signaling and upregulation of those with positive effects on telomere capping and organ morphogenesis (27). TERRA at loci co-occupied by ATRX promotes gene expression while the ATRX protein exerts the opposite function (27). Overall, TERRA molecules along with ATRX and other chromatin remodeling proteins bind to chromatin on a global scale with the highest density at chromosome ends and regulate chromatin structure and expression of hundreds of genes. Thus, TERRA is both cis- and trans-acting at telomeric sites and on the global genome, respectively (27).

### TERRA as a scaffold for shelterin subunits

TERRA also constitutes a hub for shelterin protein subunits. The nucleotide repeats of TERRA enable the formation of G-quadruplexes within TERRA itself similarly as in telomeric regions. TERRA G-quadruplexes are bound to telomeric DNA *via* the TRF2 shelterin subunit and physically interact also with TRF1 (28, 29). It has been documented that inhibition of TERRA-TRF2 interaction results in an altered localization of TERRA and induction of DDR (11). Depletion of TRF2 was shown to induce massive DDR at telomeres and formation of telomere dysfunction-induced foci (30). These cellular events result in an increased level of TERRA which in turn associates with lysine demethylase LSD1 (30). In this study, TERRA was also shown to enhance binding affinity between LSD1 and the nuclease MRE11, both crucial for the processing of uncapped telomeres (30). Another study demonstrated changes in TERRA expression and the interaction between TERRA and SUV39H1 H3K9 histone methyltransferase at damaged telomeres following TRF2 depletion. The accumulation of H3K9me3 at damaged telomeres promotes chromosome end-to-end fusion (31). The results define the critical role of TERRA during pathological telomere dysfunction events and indicate that TERRA does not function only as a scaffold for shelterin but at the same time, shelterin can affect TERRA expression as well. Thus, TERRA is critical for telomere protection, preservation of proper telomeric chromatin architecture, and prevention of inappropriate DDR events at telomeric loci (32–34).

In addition, shelterin recognizes and regulates many genes adjacent to interstitial telomere sequences (ITS) spread across the human genome by telomere looping (35–38). For example, it has been suggested that long telomeres with enriched TRF2 silence the telomerase reverse transcriptase (TERT) locus *via* Telomere Position Effect-Over Long Distances (TPE-OLD) (39). If telomeres are too short, telomere length-dependent loops are not possible, which, in turn, may increase TERT expression (39).

### TERRA and telomerase activity

TERRA was found to bind core telomerase components including the telomerase RNA template (TERC) through base pair interaction and TERT polypeptide, acting as a direct regulator of telomerase activity (40). hTERC forms several domains within its 451 nucleotides such as the scaRNA domain (binds Dyskerin), CR4/CR5 domain, and Pseudoknot/template domain which is associated with TERT polypeptide (41). The Template Region within TERC contains 3´-CAAUCCCAAUC-5´ nucleotides. Indeed, the 3′ end of TERRA is complementary to the telomerase RNA template region (25, 42), although it is currently unclear whether TERRA binds the TERC template region (40, 43). Redon et al. demonstrated that synthetic TERRA molecules containing 5´-UUAGGG-3´ repeats base pair with the TERC and also interact with telomerase catalytic subunit TERT [possibly binds to so-called anchor site in TERT (40)]. Redon et al. suggested a more complex effect of TERRA on telomerase than mere competition with telomeric DNA substrates (40).

An *in vivo* study on mouse embryonic stem cells demonstrated a 2-fold upregulation of telomerase activity following TERRA depletion (27). Furthermore, TERRA and TERC were shown to colocalize *in vivo* (27). Therefore, it has been surmised that TERRA negatively controls telomerase *in vivo* (27). *In vitro* study demonstrated that (UUAGGG)_3_ RNAs mimicking TERRA molecules inhibit telomerase activity (42). TERRA, on the other hand, was reported to promote telomerase-mediated telomere elongation in yeast *Schizosaccharomyces pombe* (44). Also, TERRA was shown to form TERRA-TLC1, a yeast telomerase RNA similar to hTERC, clusters, which are in turn recruited to short telomeres where those RNA foci help coordinate nucleation and activity of telomerase (45). However, whether the interactions between TERRA and telomerase have positive or negative effects on the activity of the latter in humans needs to be further elucidated (46).

### TERRA and its effect on telomere length

Another TERRA function is associated with direct regulation of telomere length (47, 48). As described in budding yeast *Saccharomyces cerevisiae*, TERRA transcription stimulates the 5′-3′ activity of Exonuclease 1 at chromosome ends, by which it regulates the telomere shortening rate (47).

In yeasts and telomerase-positive human cancer cells, TERRA fluctuates during the cell cycle, with the highest concentration of TERRA in the early G1 phase and clearance of TERRA from chromatin during the S/G2 phase (30). Dysregulation of the TERRA through the cell cycle was documented in ALT-positive cancer cells due to the loss of ATRX (49). Association between TERRA and telomeric chromatin *via* RAD51 DNA recombinase creates RNA : DNA hybrid structures called R-loops (50). R-loops have to be removed from chromatin upon replication of telomeric loci. Otherwise, their retention would lead to replicative stress, activation of DDR, and excessive telomere shortening (18, 31). In contrast, RNA : DNA hybrids at telomeres may induce telomere elongation *via* telomeric DNA recombination events which frequently occur in ALT-positive cancer cells. A recent study using the U2OS osteosarcoma cell line established that TERRA transcripts actively destabilize telomere integrity in ALT-positive cancer cells and that the inhibition of TERRA expression impairs the accumulation of DDR markers at telomeric sites and reduces ALT features (51). Therefore, TERRA transcripts seem to be a major trigger of ALT activity. The data suggest that TERRA transcription manipulation may be a potential therapeutic target in tumors utilizing the ALT mechanism for telomere elongation (51).

Also, based on a systematic analysis of telomere length carried out on more than 18 000 samples from many different cancer types, Barthel et al. demonstrated telomere shortening in 70% of cancer tissues compared with non-cancerous mucosa (52). The paradoxical question of telomere biology emerged from this and previous studies: Why the majority of cancer cells harbor short telomeres in spite of telomerase activation? One of the reasons is probably that telomerase activity in cancer cells enhances the level of TERRA (53) which was shown to negatively correlate with the expression of interferon-stimulated gene (ISG) (54). Therefore, an increased TERRA signal represses ISG expression and tumor growth (54). Overall, the finding suggests that there might be a connection to cancer cells harboring short telomeres as a beneficial state for tumor progression (53, 55). However, further studies exploring the role of TERRA regarding telomere homeostasis are required.

## TERRA expression

### Subtelomeres

TERRA expression is regulated and initiated from subtelomeres (48), chromosomal regions adjacent to terminal telomeric repeats (56). Thus, TERRA contains subtelomeric sequences at its 5´ end followed by canonical tracts of UUAGGG-3´ repeats transcribed from telomeres (50). Subtelomeres differ greatly in size among organisms, ranging from 10 kilobases in budding yeast to 500 kilobases in humans (48).

Putative TERRA promoter regions at multiple human subtelomeres were first identified by Azzalin et al. and by several independent studies (23, 50). One of the major transcription loci is embedded at the 20q subtelomere (26, 57). Experiments based on the ablation of approximately 8.1kb long fragment from the 20q subtelomere using the CRISPR-Cas9 method resulted in an almost complete downregulation of TERRA expression in 20q TERRA-KO U2OS osteosarcoma ALT-positive cells, telomere shortening, and the induction of massive DDR. This study was also the first to demonstrate the crucial importance of TERRA molecules for telomere homeostasis maintenance (57). Silva et al., who further elaborated on the origin of TERRA in U2OS cells, showed multiple other chromosome ends physiologically relevant for TERRA transcription. The group engineered Transcription Activator-Like Effectors ([TALEs], a plasmid based system) targeting consensus sequences located within twenty putative human subtelomeres with the purpouse to suppress TERRA expression. The group established that TERRA transcription suppression weakens ALT activity and suggested that the low level of TERRA molecules previously documented in 20q-TERRA-KO cells may, besides the 20q deletion, also arise due to short telomeres or clonal variability (51).

It is required to note that the copy number variation of the 20q13.3 subtelomeric region was identified in association with gastric (58) and sporadic colorectal cancer (59). The 20q13.3 amplification target in the tumors is most likely ADRM1, an integral plasma membrane protein involved in cell adhesion. Upregulation of ADRM1 at RNA and protein levels was reported to increase growth, proliferation, and migration in cancer cells (58). It would be interesting to analyze if other human malignancies contain similar chromosomal rearrangements. Critical information in this topic may be obtained by further studies focusing on systematic inhibition of specific TERRA promoters.

### Epigenetic modifications of subtelomeres

Subtelomeres are CpG-enriched and frequently contain heterochromatic methylation patterns of histone H3 and H4 (H3K9me3, H4K20me3). These patterns are recognized and bound by heterochromatin protein 1 (HP1) (60). A consensus on these histone marks and a subtelomere chromatin structure is missing (44). The highest concentration of CpG islands is located within two distal kilobases of subtelomeres and gradually decreases upstream towards the distal end of the chromosome. The disparity in subtelomeric methylation was revealed while examining different human cell types. Subtelomeres undergo extensive methylation during embryo development (61). Sperm cells have hypomethylated subtelomeres, while human peripheral blood leukocytes have a high level of methylation in subtelomeric regions (61). Interestingly, cancer cells, irrespectively of TMM, display variation in methylation of subtelomeric CpG islands and deregulated TERRA expression (42). Those CpG islands are, in general, heavily methylated in telomerase-positive cancer cells. This epigenetic state results in dampened TERRA expression. The maintenance of subtelomeric heterochromatin state and low TERRA levels may be therefore necessary for telomerase function in telomerase-positive tumors presumably due to the effect of TERRA on telomerase activity (62). On the other hand, ALT-positive tumor cells show, in comparison with telomerase-positive cells, heterogeneous methylation changes in subtelomeric loci and a high level of TERRA transcripts, which may be essential for the maintenance of telomeres in those cells (62).

During and after transcription, TERRA is subject to co-/post-transcriptional modifications. The RNA processing varies between individual TERRA transcripts creating biochemically different TERRA fractions with remarkably diverse biological functions (25). TERRA is transcribed by RNA Polymerase II and therefore has a canonical 7-methylguanosine cap structure at 5´ ends like most coding RNA species. Only a minor fraction of TERRA has been shown to contain poly-A tail (poly(A)+), affecting its stability and affinity to chromatin (11, 25). Poly(A)+ TERRA population is present mainly in the nucleoplasm and has a weak chromatin affinity, while poly(A)- TERRA, in addition to being located at the nucleoplasm, associates with DNA predominantly at telomeric and other chromatin sites (25).

### Regulation of TERRA expression

Expression of TERRA was shown to be regulated by major tumor suppressors (63, 64). Tutton et al. documented induction of TERRA expression upon treatment of human colorectal cancer (CRC) cells with etoposide, a drug producing DNA double-strand breaks. Notably, TERRA expression under such stress conditions is dependent on the p53 transcription factor, which recognizes the non-canonical p53 binding sites within subtelomeric regions. This binding confers transcription enhancer‐like functions and results in increased TERRA transcription. Thus, p53 provides a direct safeguard for human telomeres (64).

Furthermore, tumor suppressor Rb1 modifies telomeric chromatin architecture by regulating TERRA expression. Rb1 was demonstrated to bind human subtelomeres. Haploinsufficiency of RB1 leads to reduced TERRA levels, telomere shortening, and increased genomic instability, a common phenotypic feature of Rb1 deficient cells (i.e., osteosarcoma) (63). Additionally, Rb1 deficiency is associated with a shift in the patterns of telomeric histone modifications which, in turn, results in relaxed and unprotected chromatin (63). Overall, the non-canonical activity of Rb1 is associated with telomere homeostasis *via* regulation of TERRA expression (63).

Vohhodina et al. observed an increased TERRA expression in BRCA1-deficient cells. At telomeres and subtelomeres, BRCA1 depletion led to an altered chromatin architecture which resulted in elevated RNA Polymerase II binding to these regions. Moreover, in the absence of BRCA1, elevated R-loop levels were detected at subtelomeric CpG-island-containing TERRA promoters. Increased frequency of R-loops was associated with reduced recruitment of DNA methyltransferase, hypomethylation of TERRA promoter regions, and increased TERRA expression. Based on these observations, it can be proposed that BRCA1 regulates TERRA expression *via* the suppression of R-loop formation at subtelomeres (65).

TERRA expression is also tightly connected to cellular stress and DDR. For example, in response to heat stress, TERRA is upregulated by the heat shock factor 1 (HSF1) which was documented to bind subtelomeric regions in HeLa cell lines. Moreover, Koskas et al. detected a significantly higher frequency of DDR at telomeres in HSF1-KO cells compared to wild-type cells when cultured in the same conditions (66, 67). Interestingly, TERRA induction appeared to be a dynamic response to oxidative stress. Upon exposure to oxidative stress, TERRA expression is increased. If the stressor is removed, TERRA expression reverts after (66). Therefore, it seems that chromatin changes in subtelomeric regions displayed some sort of transcriptional memory to secure rapid expression of genes when stress was repeated.

TERRA expression is also influenced by cytoskeleton reorganization. TERRA level decreases together with decreasing surface stiffness of the cell. Cytoskeleton alterations may be produced by treatment with paclitaxel or colcemid, ultimately resulting in increased TERRA levels (67). Also, telomeres are under physiological circumstances associated with the nuclear envelope. The most recent findings on fission and budding yeast demonstrated elevated TERRA expression following detachment of telomeres from the nuclear envelope. This observation remains to be established in human cells (68). However, we can speculate whether impaired telomere-nuclear envelope interactions in humans and thereby misregulation of TERRA expression are connected with telomere-associated diseases including progeria, telomeropathies, and also cancer.

### Deregulation of TERRA expression in human pathology

Several studies have shown the association of deregulated TERRA expression with cancer. However, the role of TERRA in human solid cancers remains largely unexplored. Downregulation of TERRA along with TRF1 and upregulation of TRF2 was identified in tumor tissue of patients diagnosed with hepatocellular carcinoma (HCC) (69). Decreased level of TERRA was associated with poor prognosis of the patients and with accelerated cell growth and metastatic progression of HCC both *in vivo* and *in vitro*. Additionally, TERRA knockdown in HCC cell lines led to a significant increase in telomerase activity, telomere elongation, and increased formation of metastasis, suggesting that depleting TERRA favors the metastatic spread in HCC (69). Authors of another study found a significant reduction in TERRA expression, along with high telomerase activity and short telomeres, in endometrial cancers compared with noncancerous endometrial tissues (70). Other studies found downregulated TERRA expression in squamous cell carcinoma and astrocytoma predisposing the patients to poorer clinical outcome (71, 72). Also, in patients with astrocytoma, TERRA level correlated with the activity of telomerase, telomere length, and clinical stage (71). By contrast, upregulation of TERRA was observed in a mouse model of medulloblastoma and human cancer biopsies derived from lung, colon, ovary, breast, and stomach (73). The authors showed that TERRA concentrates in rapidly proliferating normal and cancer cells and forms foci in the nuclear regions (72). To our best knowledge, only one study has evaluated TERRA expression and the outcome of patients diagnosed with CRC. Patients with high TERRA expression and low preoperative carcinoembryonic antigen level had improved disease-free survival (74). Previous observations that telomere length may relate to cell radiosensitivity (75) were refuted by Smirnova et al. In their article, variability in TERRA levels and telomere length did not affect sensitivity to ionizing radiation in different human cell lines, including breast, gastric cancer and cervical carcinoma (76). Overall, dysregulation of TERRA was present in various human cancer tissues. TERRA was shown to accumulate and form foci in rapidly proliferating progenitor and tumor cells, supporting the presumption that TERRA expression is coupled with cell proliferation (73). The available data indicate that variation in TERRA expression across different malignancies may be tumor-type specific.

An epigenetic state of subtelomeric regions may play a critical role in TERRA expression and TMM decision too. Hypermethylated subtelomeric CpG islands in telomerase-positive cancer cell lines were detected by Nergadze et al., while demethylation of these sequences reflected in increased TERRA expression (77). In ALT-dependent cancer cells, frequent occurrence of ATRX/DAXX mutations is accompanied by DNA hypomethylation in subtelomeric regions (78). ATRX/DAXX mutations are found notably in pancreatic neuroendocrine tumors (79), and CNS malignancies (80). Depletion of ATRX and/or DAXX in the presence of various genotoxic agents is sufficient to induce ALT phenotype (81). ATRX loss induces gradual decondensation of telomere heterochromatin leading to telomeric replication stress and DDR (82). Consecutively, a cell is forced to switch to ALT to secure telomere length maintenance (82). Overall, this observation raised the possibility of distinct subtelomeric epigenetic patterns between telomerase and ALT-positive cancer cells (42).

## Cell-free TERRA as a potential diagnostic marker

Extracellular cell-free TERRA molecules (cfTERRA) have been identified in exosomes secreted into body fluids. cfTERRA is usually around 200 nucleotides in length due to post-transcriptional processing or aborted transcription from longer forms of intracellular TERRA. It has been suggested that cfTERRA levels are correlated with intracellular TERRA expression. cfTERRA is associated with histones and the binding together with high resistance to RNase contributes to cfTERRA stability and abundance in tissue and cells. Using RNA-seq analyses, cfTERRA was identified among the 20 most frequent extracellular transcripts derived from human blood plasma (83). However, the quantity of cfTERRA does not seem to be unique for malignancies, as no differences have been found between healthy subjects and various cancer patients, such as breast, colon, duct, kidney, lung, liver melanoma, ovarian, prostate, and stomach. In addition, increased cfTERRA was detected in extracellular exosomes following induced telomere dysfunction (83).

cfTERRA might belong to a family of molecules known as alarmins. These molecules (Danger Associated Molecular Pattern – DAMP; or Pathogen Associated Molecular Pattern – PAMP) are signaling cellular damage, or viral and bacterial infection (84). cfTERRA was shown to modulate the expression of the inflammatory cytokines TNFalpha and IL6 in recipient cells which represent communication between dysfunctional telomeres and inflammation through DAMP-like signaling (85). This observation may provide a mechanistic explanation of how disrupted telomere homeostasis contributes to the inflammatory cascade reaction and senescence towards neighboring cells, a bystander effect due to senescence-associated secretory phenotype (83, 86). As cfTERRA is also present at low levels in normal human plasma, it cannot stand as a single biomarker for diagnosis in itself. However, enrichment of cfTERRA along with other DAMPs might serve as a potential biomarker for the noninvasive detection of diseases associated with telomere dysfunction including cancer and telomeropathties such as familial pulmonary fibrosis, dyskeratosis congenita or aplastic anemia.

## Conclusion

In this article, we have pointed out the relevance of TERRA, a novel and exciting field of telomere biology, in the context of human physiological and pathological processes. Understanding TERRA functions is of great interest in basic medicine as TERRA regulation is altered in human diseases including cancer. However, the expression level of TERRA varies in a tumor type-specific manner (80). Based on the current knowledge, TERRA is a potential therapeutic target in different malignancies. Further studies are needed to clarify whether cancer cells harboring different TMMs have diverse methylation of subtelomeres and different patterns of TERRA expression.

Increased cfTERRA have been detected in response to telomere dysfunction, suggesting its potential use as a biomarker for the detection of early stages of cancers and other telomere-driven diseases. It is also important to further elucidate the crosstalk between cfTERRA, inflammation, and tumor microenvironment.

Overall, a deeper insight into TERRA regulation could help us understand its role in telomere maintenance and genome stability.

## Author contributions

Conceptualization: MKr; Funding acquisition: LV, MKr and KT; Visualization: PS; Writing and original draft: MKr, KT, and MKa; Review and editing: PV, RK, and TB. All authors contributed to the article and approved the submitted version.

## Funding

This study was supported by the Grant Agency of the Ministry of Health of the Czech Republic (NU22J-03-00028), the Grant Agency of the Charles University (project GA UK No. 120), and the Grant Agency of the Czech Republic (21-27902S).

## Conflict of interest

The authors declare that the research was conducted in the absence of any commercial or financial relationships that could be construed as a potential conflict of interest.

## Publisher’s note

All claims expressed in this article are solely those of the authors and do not necessarily represent those of their affiliated organizations, or those of the publisher, the editors and the reviewers. Any product that may be evaluated in this article, or claim that may be made by its manufacturer, is not guaranteed or endorsed by the publisher.
